# Computational Models of Reactive Oxygen Species as Metabolic Byproducts and Signal-Transduction Modulators

**DOI:** 10.3389/fphar.2016.00457

**Published:** 2016-11-29

**Authors:** Elizabeth J. Pereira, Christian M. Smolko, Kevin A. Janes

**Affiliations:** Department of Biomedical Engineering, University of Virginia, CharlottesvilleVA, USA

**Keywords:** systems biology, oxidative stress, superoxide, NAPDH oxidase, cancer, cardiovascular

## Abstract

Reactive oxygen species (ROS) are widely involved in intracellular signaling and human pathologies, but their precise roles have been difficult to enumerate and integrate holistically. The context- and dose-dependent intracellular effects of ROS can lead to contradictory experimental results and confounded interpretations. For example, lower levels of ROS promote cell signaling and proliferation, whereas abundant ROS cause overwhelming damage to biomolecules and cellular apoptosis or senescence. These complexities raise the question of whether the many facets of ROS biology can be joined under a common mechanistic framework using computational modeling. Here, we take inventory of some current models for ROS production or ROS regulation of signaling pathways. Several models captured non-intuitive observations or made predictions that were later verified by experiment. There remains a need for systems-level analyses that jointly incorporate ROS production, handling, and modulation of multiple signal-transduction cascades.

## Introduction

Reactive oxygen species (ROS) play a complex role in cellular biology. Initially viewed merely as harmful byproducts of metabolism, ROS are now known to serve additional functions as intracellular regulators of various signaling pathways. At low levels, ROS function as a reactive second messenger mainly by reversible oxidation of key amino acids of target proteins. High ROS levels, by contrast, cause damage to various biomolecules (lipids, proteins, and DNA) and have been linked to pathologies including neurodegeneration (Smith et al., 1997; Giasson et al., 2000), atherosclerosis (Patetsios et al., 2001; Guzik et al., 2006), and renal disease (Nishikawa et al., 2000; Dounousi et al., 2006). ROS play an especially complex role in cancer, with various oncogenes and tumor suppressors influencing, and influenced by, the redox environment of the cell (Meng et al., 2002; Leslie et al., 2003).

It is challenging to study experimentally how endogenous and exogenous sources of ROS are handled by the cell. In addition, the context- and dose-dependent intracellular consequences of ROS can result in confounding observations. ROS has been found to stimulate proliferation in some cell types under certain experimental conditions (Ruiz-Ginés et al., 2000; Nieto et al., 2002) and inhibit proliferation (Qu et al., 2011) or induce apoptosis (Pierce et al., 1991) in others. Such contextual and experimental complexities make it difficult to understand ROS holistically by experimentation alone.

**FIGURE 1:**
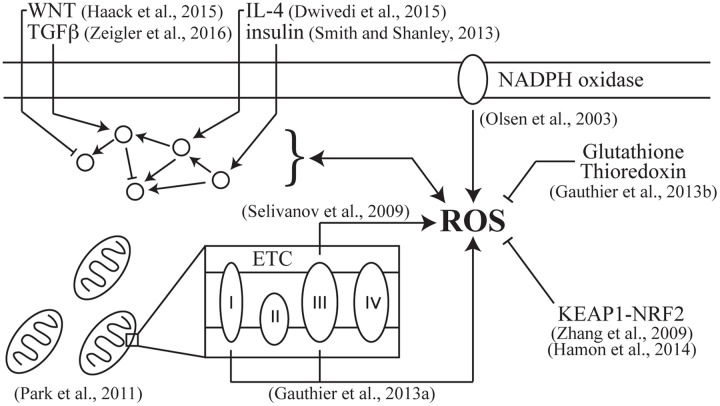
**Computational models of reactive oxygen species (ROS) production and signal-transduction modulation**.

Computational modeling approaches can tackle this problem by simulating the concurrent dynamics of many variables, including those that are difficult to access experimentally (Janes and Lauffenburger, 2013). Our review here covers the handful of models described thus far for ROS production and ROS regulation of signaling pathways (**Figure 1**). We start with a brief introduction of ROS, the biological processes that generate them, and the signaling pathways that sense ROS and detoxify the cell. We next focus on models that simulate ROS production by the mitochondria, the predominant intracellular source of ROS, and by membrane-bound enzymes, which link extracellular signaling to intracellular ROS production. We also discuss models that simulate ROS regulation of various signaling pathways, giving a broader view of the influence ROS have on intracellular signaling. Finally, we discuss the need for a systems-level analysis of ROS signaling to provide a generalizable framework that accounts for the many downstream cellular effects of ROS.

## ROS: Sources, Scavengers, and Effectors

Reactive oxygen species are chemically reactive species derived from the incomplete reduction of oxygen. Common examples are hydrogen peroxide (H_2_O_2_), superoxide ($O_{2}^{-}$), and hydroxyl radicals (HO⋅). For most cell types, ROS production mainly occurs through mitochondrial oxidative phosphorylation. Inherent leakiness of the electron transport chain (ETC), specifically from complex I and complex III, causes some electrons to flow out of the pathway and partially reduce oxygen to the superoxide anion. Other endogenous sources of ROS include the membrane-bound enzyme NADPH oxidase, which produces ROS in response to various ligands, along with other enzymes such as xanthine oxidase, cyclooxygenases, and nitric oxide synthase. To detoxify excess ROS, cells are equipped with antioxidants, including the thiols glutathione and thioredoxin, which are regulated by the transcription factor NRF2 (Itoh et al., 1997; Chorley et al., 2012). Tight regulation of ROS by antioxidant systems is necessary to balance the generation of ROS and to curtail oxidative damage to biomolecules.

## Challenges of Measuring ROS

Reactive oxygen species are difficult to measure reliably within cells. For example, early measurements of cellular oxidative state used dyes such as 2′,7′-dichlorofluorescin (DCFH) which were later shown to create additional radical species (Rota et al., 1999). The secondary reactions and instability of the dye made long-term imaging with DCFH impossible. To address these deficiencies, researchers engineered fluorescent reporter proteins such as HyPer, RoGFP, and RxYFP (Hanson et al., 2004; Belousov et al., 2006; Meyer and Dick, 2010), which undergo redox-sensitive conformational changes that elicit a change in fluorescence. Genetically encoded fluorescent proteins enable live-cell imaging and are further capable of localizing ROS production to specific sub-cellular compartments (Malinouski et al., 2011; Swain et al., 2016). Nevertheless, these sensors might miss low concentrations of H_2_O_2_ due to the endogenous enzyme peroxiredoxin, which is ∼100-fold more active toward H_2_O_2_ compared to introduced probes (Ezeriņa et al., 2014).

Given these challenges, some groups have taken a more computational approach to calculating the kinetics associated with H_2_O_2_ (Brito and Antunes, 2014). Lim et al. built a reaction-diffusion model to study localization of H_2_O_2_, which is important for control and specificity of redox signaling. They incorporated cytoplasmic diffusion into their reduced kinetic model of H_2_O_2_ clearance, in which peroxiredoxin is the dominant scavenging molecule (Lim et al., 2015). Using modeled concentration profiles obtained after bolus addition of H_2_O_2_ to the extracellular medium, the authors determined order-of-magnitude estimates for intracellular H_2_O_2_ diffusion through the cytosol, with a length scale of 4 μm and a time scale of 1 ms (Lim et al., 2016). The short length scale and rapid time scale indicate that H_2_O_2_ degradation and signaling are localized to the area where H_2_O_2_ is produced, contradicting the common modeling assumption of a well-mixed cytoplasm. This finding could explain discrepancies observed between bolus addition versus steady intracellular generation of H_2_O_2_ (Sobotta et al., 2013; Cheong et al., 2015). Rapid H_2_O_2_ scavenging also has implications for intracellular signaling, as H_2_O_2_ reactivity is limited to molecules in the subcellular vicinity.

## ROS Production By Complex III of the ETC

Physiologically, ROS production increases under hypoxic conditions. Hypoxia causes a decrease in the maximum reaction rate of complex IV, which is thought to cause excess electron leakage from other components of the ETC, such as complex III (Chandel et al., 2000). After a return to normoxic conditions, ROS remain at higher hypoxic levels. The stable switch in ROS production is relevant during organ transplantation and other surgeries requiring an ischemic period. To better understand the mechanism behind this bistability, Selivanov et al. (2009) modeled the Q cycle mechanism of Complex III in the mitochondrial respiratory chain as the primary mechanism of ROS generation. Complex III can take on as many as 400 redox states due to its binding to quinones. The authors elaborated a system of differential equations describing the evolution of all of the redox states of Complex III. Model simulations predicted that Complex III can exist in two different steady states, a low ROS-producing state and a high ROS-producing state. This bistability is dependent upon the initial conditions of the system, specifically the redox state as predicted by levels of semiquinone and free ubiquinol. If starting in a highly reduced state, the overall system remains reduced, whereas if it starts in a less reduced state, Complex III progresses to a steady state with low semiquinone concentration and thus low ROS production. The overall system evolves to the high ROS producing state either by an increase in succinate concentration, causing the reduction of ubiquinone to ubiquinol, or a decrease in oxygen content. Once switched to this high ROS-producing state, Complex III persists in that state even after a return to lower succinate concentration or normoxic conditions. The sustained increase in ROS production provides a mechanism that may contribute to reperfusion injury after ischemia.

The model predictions were experimentally validated in isolated rat brain mitochondria incubated with succinate with or without the addition of ADP. The addition of ADP, and subsequent synthesis of ATP, switches mitochondria to a low ROS-producing state and thereby lowers mitochondrial membrane potential. Once all the ADP is consumed, membrane potential increases to pre-ADP levels, but ROS production remains at the lower initial level. These results agree with model predictions that two levels of ROS production could coexist under the same set of parameters and give rise to metabolic heterogeneity in an isogenic population of cells.

## ROS Production By Complexes I and III of the ETC

Reactive oxygen species are also produced by Complex I of the ETC. Gauthier et al. (2013a) built a computational model of the ETC focusing on ROS production by both complex I and III (**Figure 1**). Simulations were used to study the control of ROS production in cardiac myocytes under different metabolic conditions. The model is composed of non-linear ordinary differential equations describing the oxidation states of the various forms of ubiquinone, produced by complex I electron transfer, and the three subunits of complex III: cytochrome b, cytochrome c1, and the iron sulfur protein. The authors investigated how mitochondrial membrane potential, matrix pH, and ROS scavenging affect ROS production and control. When membrane potential increased 20 mV higher than unstressed cells to above ∼150 mV (Padmaraj et al., 2014), the model predicted that complex III ROS production as a function of membrane potential switches from zeroth order (constant production) to first order (exponential production). Increased membrane potential leads to a reduction in the Q cycle reaction rate, or conversion of ubiquinol to ubiquinone, causing a substantial increase in ROS production rate.

The model predictions agree with experimental results reporting a threshold membrane potential of 153 mV, above which ROS production increases dramatically (Korshunov et al., 1997). When simulating an increase in mitochondrial matrix pH, the model predicted that ROS production from complexes I and III increases during forward electron transport. This pH-dependent mechanism of ROS generation was experimentally observed by Selivanov et al. (2008) who found that an increase in pH from 6 to 7 caused a threefold increase in ROS production rate. During reverse electron transport, where electrons flow toward complex I in the presence of a weak reducing agent, complex I ROS production also increased with matrix alkalinization. The model therefore correctly predicted the dependence of ROS production on both mitochondrial membrane potential and matrix pH.

ROS levels are governed not only by production, but also by clearance through scavenging mechanisms. To gain a more complete picture of ROS dynamics, Gauthier et al. added glutathione and thioredoxin-mediated ROS scavenging mechanisms to the model (Aon et al., 2012). ROS production decreased to a minimum level as the mitochondrial environment became more oxidized and then rose again as the scavenging systems became depleted. This result agrees with the redox-optimized ROS balance hypothesis (Aon et al., 2010), which states that ROS levels are lowest at an intermediate mitochondrial redox potential. Together, the authors’ minimal model of ROS regulation produced results that matched many independent experiments describing different regimes of ROS production, providing support for the hypothesis that the cellular redox state influences the rate of ROS production.

Applying their model, Gauthier went on to investigate ROS production and scavenging in the context of heart failure (Gauthier et al., 2013b). Integrating the ETC-ROS model discussed above into a mitochondrial energetic-redox model (Kembro et al., 2013) allowed the authors to test the hypothesis that mitochondrial Ca^2+^ mismanagement leads to high levels of ROS during heart failure. In agreement with this hypothesis, their model showed that under conditions of mismanaged mitochondrial Ca^2+^, NADH levels decrease drastically under simulated cardiac pacing, highlighting the link between compromised Ca^2+^ and NADH regulation. Lower amounts of NADH lead to lower NADPH levels and a decreased ability to reduce scavenging enzymes for reuse, causing ROS to accumulate. ROS levels in mitochondria increase under increased load (Satapati et al., 2015), but surprisingly the model predicted that ROS production actually decreases under these conditions. The net increase in ROS abundance stems from an even-further reduction in ROS scavenging, which causes ROS accumulation in the cell. Therefore, in the setting of heart failure, preservation or restoration of scavenging enzymes may prove more effective than efforts to block ROS production (Linke et al., 2005).

In another model of ROS production by ETC complexes I and III, Bazil et al. (2016) described ROS generation by oxidative phosphorylation coupled to ATP demand. They updated an existing kinetic model of oxidative phosphorylation (Beard, 2005) to include ROS generation by complexes I and III and first-order scavenging by superoxide dismutase and peroxidase. Model simulations agreed with previous findings that free radical production by complex III is higher than complex I production under physiological conditions (Gauthier et al., 2013a). As ATP demand increases, the steady state production of ROS also increases, in line with experimental observations (Liu, 2010). The authors further applied their model to study reverse electron transport that is seen during reperfusion. Simulating ischemia/reperfusion led to bistability in ROS production (Selivanov et al., 2009, 2011) only when the activity of complex II was increased. Complex II activity requires electrons to be supplied to the quinone pool by the dehydrogenation of succinate to fumarate. The predicted importance of complex II agrees with work by Chouchani et al. (2014) showing that succinate is a main driver of mitochondrial ROS production upon reperfusion (Chouchani et al., 2014). Therefore, complex II inhibition during reperfusion could prove useful to decrease ROS production and reperfusion injury (Valls-Lacalle et al., 2016).

## ROS Production By the Mitochondrial Network

In a phenomenon known as ROS-Induced ROS Release (RIRR), damaged mitochondria produce an increased amount of ROS, which causes surrounding mitochondria to increase ROS production through a positive-feedback loop. Park et al. (2011) used an agent-based model describing inter-mitochondrial signaling to study the role of mitochondrial network dynamics in mitochondria-driven ROS production (**Figure 1**). Simulations were performed with three different mitochondrial networks: uniformly distributed mitochondria, as seen in cardiomyocytes; irregularly distributed mitochondria, as in neurons; and sparsely distributed mitochondria, as found in white blood cells (Maianski et al., 2004). The simulations introduced hydrogen peroxide as an initial oxidative stress, causing mitochondria in the surrounding area to produce more ROS. Mitochondrial ROS diffuse stochastically by random walk in 2D space, amplifying the local ROS response. Depending on the initial oxidative stress insult and the mitochondrial network dynamics, ROS production is blocked by antioxidant enzyme systems or becomes amplified by RIRR, which propagates ROS through the entire cell. The goal of the model was to predict the percent reactive mitochondria resulting from an initial oxidative stress input and the initial hydrogen peroxide concentration that causes RIRR.

The model indicated that ROS propagation is faster in the cardiomyocyte model than in the irregular distribution model, as shown by a higher dose dependence of reactive mitochondria as a function of initial oxidative stress. In addition to mitochondrial distribution, the model predicted that the density of mitochondria affects the response to oxidative stress inputs. Cells with a low density of mitochondria have considerable ROS propagation after low levels of oxidative stress, while cells with a high density of mitochondria only show strong ROS propagation after high levels of oxidative stress. The authors hypothesized that these differing responses to oxidative stress are due to differences in ROS signal transduction between mitochondrial networks. They further simulated the addition of different antioxidants to find that superoxide-scavenging antioxidants block ROS propagation more effectively in the cardiomyocyte model, while antioxidants that detoxify hydrogen peroxide are more effective in the irregular-distribution and low-density models of mitochondria. These results suggest that mitochondrial network configuration influences which molecular species is used to propagate ROS in the cell.

## ROS Production in Relation to Antioxidant Signaling

Cyclosporin A (CsA) is an immunosuppressant, which indirectly causes oxidative stress (Parra Cid et al., 2003) and adaptively activates the NRF2 pathway in the kidney. Hamon et al. (2014) fused an *in vitro* pharmacokinetic model (Wilmes et al., 2013) of CsA distribution in cultured renal epithelial cells with a dynamical model of NRF2 signaling originally designed to capture the cellular response to xenobiotics (Zhang et al., 2009). The authors adapted the NRF2 model to accommodate ROS as a state variable generated in proportion to cytosolic CsA (Hamon et al., 2014). In the revised model, ROS are detoxified by glutathione peroxidase and further act as an oxidant and inhibitor of KEAP1, which degrades NRF2. Last, CsA was forbidden from interacting with the aryl hydrocarbon receptor as xenobiotics do in the original model, because experimental data was lacking for such an interaction. To parameterize the fused model, Bayesian inference was used together with transcriptomic, proteomic, and metabolomic data collected from cells treated with different concentrations of CsA dosed daily for 2 weeks. The model predicted that low doses of CsA yielded widespread oscillations throughout the network as cells metabolized the administered CsA and detoxified ROS before the next administration. At high doses, however, the cell is overwhelmed and the modeled network locks into an elevated state of ROS adaptation. These predictions were not followed up experimentally, but the work of Hamon et al. (2014) nonetheless illustrates how toxicologic models can be repurposed for ROS specifically.

## ROS Production in the Phagosome Membrane By NADPH Oxidase

Aside from the ETC, ROS also play a key role in pathogen clearance. Neutrophils utilize ROS to attack bacteria engulfed within a phagosome. The source of this ROS burst is not from mitochondrial respiration but from the NADPH oxidase complex at the plasma membrane (Henderson and Chappel, 1996) (**Figure 1**). Levels of ROS oscillate in the neutrophil (Wymann et al., 1989); however, the mechanism behind these oscillations was unclear. Olsen et al. (2003) proposed that the oscillations arose from interactions among myeloperoxidase, melatonin, NADPH, and NADPH oxidase. To explore the oscillatory behavior, they built a two-compartment, differential equation model of the phagosome and the cytoplasm (Olsen et al., 2003). Without NADPH oxidase activity, model simulations exclusively produced damped oscillations that converged to a steady-state; by contrast, addition of NADPH oxidase elicited sustained oscillations similar to those reported experimentally (Petty, 2001). The authors triggered ROS oscillations in neutrophils with the activating chemotactic peptide FMLP and showed that pre-incubation with an inhibitor of NADPH oxidase blocked oscillations. Their model further predicted that melatonin would change the amplitude of the ROS oscillations measured. Pre-incubation of FMLP-activated neutrophils with melatonin confirmed the predicted increases in ROS amplitude. Computational and experimental modeling of NADPH oxidase in this setting allowed the authors to understand the basis of melatonin “priming” previously observed in neutrophils (Recchioni et al., 1998), underlining the power of pairing *in silico* and *in vitro* experiments.

## ROS Production During WNT/β-Catenin Signaling

Reactive oxygen species are also generated as a secondary byproduct of multiple signal transduction cascades (Meier et al., 1989; Thannickal and Fanburg, 1995). Haack et al. (2015) built a model of the WNT/β-catenin signaling pathway that included membrane-related processes as well as ROS signaling. The authors sought to explain experimental results showing that disruption of membrane lipid rafts inhibits WNT/β-catenin signaling, and also that ROS activate WNT signaling in the context of differentiation of human neural progenitor cells (Funato et al., 2006; Rharass et al., 2014). The three-compartment model is based on mass-action kinetics and includes: a membrane model, in which WNT binds to receptor LRP6 causing its phosphorylation within lipid rafts; an intracellular model, in which AXIN binds phosphorylated LRP6 to prevent degradation of β-catenin; and a redox model, in which ROS increase the concentration of DVL-bound AXIN, making AXIN unable to degrade β-catenin. Simulations were initiated with a burst of ROS that was shown experimentally to coincide with the beginning of neural progenitor differentiation induced by growth-factor withdrawal. The model predicted an immediate, transient β-catenin stabilization resulting from redox-dependent DVL/AXIN binding, followed by a sustained β-catenin response arising from lipid raft-dependent canonical WNT signaling. The immediate β-catenin accumulation was observed experimentally by the authors in lipid raft-deficient cells that maintained a transient β-catenin response. By including ROS signaling, the extended WNT/β-catenin signaling model correctly captured experimental β-catenin nuclear dynamics during early neuronal differentiation.

## ROS Modulation of IL-4 Signaling

Dwivedi et al. (2015) looked at modulation of cell signaling by ROS in another setting, using the IL-4 signaling pathway as a redox-regulated case study. The authors sought to identify the most important mechanisms of redox regulation in the IL-4 pathway, which is important for regulating the effector T-cell response. The activated IL-4 receptor complex upregulates ROS through NADPH oxidase (Sharma et al., 2008), which influences signal transduction that proceeds through JAKs and culminates in the phosphorylation of STAT6. To identify the combination of regulatory mechanisms that best recapitulated the dynamics of IL-4 induced STAT6 phosphorylation, the authors turned to Monte Carlo analysis of an IL-4 ordinary differential equation model. Phosphorylated STAT6 dynamics were best captured by a model that incorporated a protein tyrosine phosphatase whose activity and nucleocytoplasmic shuttling were ROS sensitive. ROS regulation of phosphatase activity and localization, along with other ROS-independent mechanisms, were included in the systems-level model of IL-4 signaling, with parameters fit to experimental data in IL-4-stimulated Jurkat cells. The model predicted diminished STAT6 phosphorylation following IL-4 stimulation and ROS inhibition, which was confirmed experimentally by NADPH oxidase inhibition of IL-4-stimulated cells. Transient oxidation of protein tyrosine phosphatases was also observed experimentally by oxidized protein tyrosine phosphatase immunoprecipitation of extracts from IL-4-stimulated Jurkat cells. The authors’ systems-level model provides a framework for investigating additional modes of receptor-initiated oxidation not previously explored.

## ROS Crosstalk with Insulin Signaling

Smith and Shanley (2013) adapted an existing differential equation model of insulin signaling (Sedaghat et al., 2002) to incorporate ROS and study the interplay between insulin signaling and oxidative stress (**Figure 1**). Insulin-stimulated ROS production was assumed to occur through activation of NADPH oxidase and is about fivefold higher than the background level of mitochondrially produced ROS (Mahadev et al., 2001). ROS deactivate the phosphatases PTEN and PTP, activate the kinases JNK and IKK, and are detoxified by cytoplasmic SOD2. This model was used to make predictions about ROS, FOXO, SOD2, and insulin receptor abundances over long time scales.

When hydrogen peroxide was added as an oxidant to the system with or without insulin stimulation, the model predicted surprisingly different responses. Hydrogen peroxide alone caused modest glucose uptake and insulin alone caused strong glucose uptake, but hydrogen peroxide and insulin stimulation together were antagonistic, causing only moderate glucose uptake. In the model, this dampening effect of oxidative stress occurs because hydrogen peroxide and insulin together activate protein kinases (e.g., JNK, IKK), which cause hyperphosphorylation of IRS1 and decrease its ability to form the IRS1-PI3K complex that stimulates glucose uptake. The model also predicted that the FOXO-mediated antioxidant response depends critically on the extent of oxidative stress. With low oxidative stress, the antioxidant enzyme SOD2 is upregulated by FOXO through a JNK-mediated mechanism, but SOD2 is downregulated at higher levels of stress through an IKK-mediated mechanism. Although simplified in its handling of ROS and antioxidant pathways, this integrated systems model captures some of the complexities of oxidative stress for an important metabolic pathway.

## ROS Production As One Node in A Larger Network of Cardiac Fibroblast Signaling

The previously discussed models incorporated ROS into a single canonical signaling pathway, but the generation and handling of ROS pervades multiple pathways and can lead to counterintuitive cell outcomes (Wang et al., 2011). Zeigler et al. (2016) incorporated ROS as part of a much larger signaling network to identify regulators of cardiac fibrosis. A cardiac fibroblast signaling network was designed to study drivers of fibrosis, which is implicated in many cardiac pathologies (Moreo et al., 2009). The network was compiled from experimental data on 10 pathways that are known to be important in cardiac injury, such as the IL-1 and TGFβ pathways. The model was formed using a logic-based differential equation approach (Kraeutler et al., 2010), whereby species are represented as differential equations with rates of change dictated by Hill functions and truth tables comprised of interacting biomolecules. ROS production is controlled by the activity of NADPH oxidase and feeds into the truth tables of JNK and ERK activation, linking ROS generation at the plasma membrane to downstream intracellular responses.

In the model, reducing ROS levels had far-reaching and context-dependent effects on the network. Under baseline conditions, reductions in ROS caused a decrease in matrix metalloproteinase 9 (MMP9), which is important for the breakdown of extracellular matrix. By contrast, in an environment with high TGFβ signaling, like in a myocardial infarction, reducing ROS led to an increase in MMP9 activity. Therefore, a therapeutic prediction of the model is that antioxidant treatment for fibrosis would be more beneficial in the high TGFβ environment of myocardial infarct.

## Conclusion and Future Outlook

The computational models of ROS biology covered in this review largely focus on ROS handling within the cell or on ROS modulation of canonical signaling pathways. In the future, we anticipate more sophisticated models that combine ROS handling and signaling concurrently. A prime test bed for such an integrated approach would be the NF-κB pathway, which is activated by ROS (Schreck et al., 1991) and is responsible for inducing scavenging enzymes such as SOD2 (Wong and Goeddel, 1988). Finn and Kemp (2012) have assembled a provisional model of IKKβ S-glutathionylation in the setting of antioxidants and chemotherapy-induced ROS. The coupling of signaling, production, and scavenging could give rise to feedback networks that explain the variable oxidative stress observed in some settings among single cells in very similar microenvironments (Wang et al., 2011).

There is also a need to build multiscale models that place ROS in the broader context of developing tissues, tumors, and infections (Schwarz, 1996; Bajikar and Janes, 2012; Wang et al., 2012). The dynamics of proliferation and death impinge on metabolism and signal transduction, which culminate to impact the redox state of cells in the population. Crosstalk between these cellular pathways may require different classes of modeling than those implemented so far (Anderson et al., 2006; Chitforoushzadeh et al., 2016). Advances in measurement will likewise expand the scope of targets modified by ROS (Kim et al., 2015) and reveal the extent to which molecular crosstalk is underappreciated.

Integration of ROS signaling into larger networks may allow researchers to predict outcomes of drug treatments that affect ROS generation, which causes drug resistance in some cancer contexts (Nieborowska-Skorska et al., 2014; Okon et al., 2015). A deeper understanding of ROS network dynamics could generate combinatorial treatments that avoid neutralizing drug efficacy. In the broader human population, there are many polymorphisms that affect ROS generation and scavenging, such as p22^phox^ C242T and SOD2 A16V (Bresciani et al., 2013; Meijles et al., 2016). These variants may tune how ROS interacts with other signaling networks, contributing to heterogeneous patient responses during therapy.

ROS are a fact of life that cannot be ignored. Like a living cell, investigators must find ways to deal with ROS holistically and achieve our goals despite their presence. The tools for pharmacologic modulation of ROS are predominantly limited to antioxidants. Systems modeling of ROS may one day provide a venue for exploring more-precise interventions that account for the complex biological processes involved.

## Author Contributions

EP and CS conceived of the review with input from KJ. EP and CS drafted the review with revisions provided by KJ.

## Conflict of Interest Statement

The authors declare that the research was conducted in the absence of any commercial or financial relationships that could be construed as a potential conflict of interest.

## References

[B1] Anderson A. R. A. Weaver A. M. Cummings P. T. Quaranta V. (2006). Tumor morphology and phenotypic evolution driven by selective pressure from the microenvironment. *Cell* 127 905–915. 10.1016/j.cell.2006.09.042 17129778

[B2] Aon M. A. Cortassa S. O’Rourke B. (2010). Redox-optimized ROS balance: a unifying hypothesis. *Biochim. Biophys. Acta* 1797 865–877. 10.1016/j.bbabio.2010.02.016 20175987 PMC2891851

[B3] Aon M. A. Stanley B. A. Sivakumaran V. Kembro J. M. O’Rourke B. Paolocci N. (2012). Glutathione/thioredoxin systems modulate mitochondrial H2O2 emission: an experimental-computational study. *J. Gen. Physiol.* 139 479–491. 10.1085/jgp.201210772 22585969 PMC3362521

[B4] Bajikar S. S. Janes K. A. (2012). Multiscale models of cell signaling. *Ann. Biomed. Eng.* 40 2319–2327. 10.1007/s10439-012-0560-1 22476894 PMC3436998

[B5] Bazil J. N. Beard D. A. Vinnakota K. C. (2016). Catalytic coupling of oxidative phosphorylation, ATP demand, and reactive oxygen species generation. *Biophys. J.* 110 962–971. 10.1016/j.bpj.2015.09.036 26910433 PMC4776027

[B6] Beard D. A. (2005). A biophysical model of the mitochondrial respiratory system and oxidative phosphorylation. *PLoS Comput. Biol.* 1:e36 10.1371/journal.pcbi.0010036 PMC1201326 16163394

[B7] Belousov V. V. Fradkov A. F. Lukyanov K. A. Staroverov D. B. Shakhbazov K. S. Terskikh A. V. (2006). Genetically encoded fluorescent indicator for intracellular hydrogen peroxide. *Nat. Methods* 3 281–286. 10.1038/nmeth866 16554833

[B8] Bresciani G. Cruz I. B. M. de Paz J. A. Cuevas M. J. González-Gallego J. (2013). The MnSOD Ala16Val SNP: relevance to human diseases and interaction with environmental factors. *Free Radic. Res.* 47 781–792. 10.3109/10715762.2013.836275 23952573

[B9] Brito P. M. Antunes F. (2014). Estimation of kinetic parameters related to biochemical interactions between hydrogen peroxide and signal transduction proteins. *Front. Chem* 2:82 10.3389/fchem.2014.00082 PMC4183122 25325054

[B10] Chandel N. S. McClintock D. S. Feliciano C. E. Wood T. M. Melendez J. A. Rodriguez A. M. (2000). Reactive oxygen species generated at mitochondrial complex III stabilize hypoxia-inducible factor-1alpha during hypoxia: a mechanism of O2 sensing. *J. Biol. Chem.* 275 25130–25138. 10.1074/jbc.M001914200 10833514

[B11] Cheong T.-C. Shin E. P. Kwon E.-K. Choi J.-H. Wang K.-K. Sharma P. (2015). Functional manipulation of dendritic cells by photoswitchable generation of intracellular reactive oxygen species. *ACS Chem. Biol.* 10 757–765. 10.1021/cb5009124 25458073

[B12] Chitforoushzadeh Z. Ye Z. Sheng Z. LaRue S. Fry R. C. Lauffenburger D. A. (2016). TNF-insulin crosstalk at the transcription factor GATA6 is revealed by a model that links signaling and transcriptomic data tensors. *Sci. Signal.* 9 ra59 10.1126/scisignal.aad3373 PMC4914393 27273097

[B13] Chorley B. N. Campbell M. R. Wang X. Karaca M. Sambandan D. Bangura F. (2012). Identification of novel NRF2-regulated genes by ChIP-Seq: influence on retinoid X receptor alpha. *Nucleic Acids Res.* 40 7416–7429. 10.1093/nar/gks409 22581777 PMC3424561

[B14] Chouchani E. T. Pell V. R. Gaude E. Aksentijević D. Sundier S. Y. Robb E. L. (2014). Ischaemic accumulation of succinate controls reperfusion injury through mitochondrial ROS. *Nature* 515 431–435. 10.1038/nature13909 25383517 PMC4255242

[B15] Dounousi E. Papavasiliou E. Makedou A. Ioannou K. Katopodis K. P. Tselepis A. (2006). Oxidative stress is progressively enhanced with advancing stages of CKD. *Am. J. Kidney Dis.* 48 752–760. 10.1053/j.ajkd.2006.08.015 17059994

[B16] Dwivedi G. Gran M. A. Bagchi P. Kemp M. L. (2015). Dynamic Redox Regulation of IL-4 Signaling. *PLoS Comput. Biol.* 11:e1004582 10.1371/journal.pcbi.100458 PMC4642971 26562652

[B17] Ezeriņa D. Morgan B. Dick T. P. (2014). Imaging dynamic redox processes with genetically encoded probes. *J. Mol. Cell. Cardiol.* 73 43–49. 10.1016/j.yjmcc.2013.12.023 24406687

[B18] Finn N. A. Kemp M. L. (2012). Pro-oxidant and antioxidant effects of N-acetylcysteine regulate doxorubicin-induced NF-kappa B activity in leukemic cells. *Mol. Biosyst.* 8 650–662. 10.1039/c1mb05315a 22134636 PMC3337722

[B19] Funato Y. Michiue T. Asashima M. Miki H. (2006). The thioredoxin-related redox-regulating protein nucleoredoxin inhibits Wnt-beta-catenin signalling through dishevelled. *Nat. Cell Biol.* 8 501–508. 10.1038/ncb1405 16604061

[B20] Gauthier L. D. Greenstein J. L. Cortassa S. O’Rourke B. Winslow R. L. (2013a). A computational model of reactive oxygen species and redox balance in cardiac mitochondria. *Biophys. J.* 105 1045–1056. 10.1016/j.bpj.2013.07.006 23972856 PMC3752118

[B21] Gauthier L. D. Greenstein J. L. O’Rourke B. Winslow R. L. (2013b). An integrated mitochondrial ROS production and scavenging model: implications for heart failure. *Biophys. J.* 105 2832–2842. 10.1016/j.bpj.2013.11.007 24359755 PMC3882515

[B22] Giasson B. I. Duda J. E. Murray I. V. J. Chen Q. Souza J. M. Hurtig H. I. (2000). Oxidative damage linked to neurodegeneration by selective α-synuclein nitration in synucleinopathy lesions. *Science* 290 985–989. 10.1126/science.290.5493.985 11062131

[B23] Guzik T. J. Sadowski J. Guzik B. Jopek A. Kapelak B. Przybylowski P. (2006). Coronary artery superoxide production and nox isoform expression in human coronary artery disease. *Arterioscler. Thromb. Vasc. Biol.* 26 333–339. 10.1161/01.ATV.0000196651.64776.51 16293794

[B24] Haack F. Lemcke H. Ewald R. Rharass T. Uhrmacher A. M. (2015). Spatio-temporal model of endogenous ROS and raft-dependent WNT/Beta-catenin signaling driving cell fate commitment in human neural progenitor cells. *PLoS Comput. Biol.* 11:e1004106 10.1371/journal.pcbi.1004106 PMC4368204 25793621

[B25] Hamon J. Jennings P. Bois F. Y. (2014). Systems biology modeling of omics data: effect of cyclosporine a on the Nrf2 pathway in human renal cells. *BMC Syst. Biol.* 8:76 10.1186/1752-0509-8-76 PMC4089556 24964791

[B26] Hanson G. T. Aggeler R. Oglesbee D. Cannon M. Capaldi R. A. Tsien R. Y. (2004). Investigating mitochondrial redox potential with redox-sensitive green fluorescent protein indicators. *J. Biol. Chem.* 279 13044–13053. 10.1074/jbc.M312846200 14722062

[B27] Henderson L. M. Chappel J. B. (1996). NADPH oxidase of neutrophils. *Biochim. Biophys. Acta* 1273 87–107. 10.1016/0005-2728(95)00140-9 8611594

[B28] Itoh K. Chiba T. Takahashi S. Ishii T. Igarashi K. Katoh Y. (1997). An Nrf2/small Maf heterodimer mediates the induction of phase II detoxifying enzyme genes through antioxidant response elements. *Biochem. Biophys. Res. Commun.* 236 313–322. 10.1006/bbrc.1997.6943 9240432

[B29] Janes K. A. Lauffenburger D. A. (2013). Models of signalling networks - what cell biologists can gain from them and give to them. *J. Cell. Sci.* 126 1913–1921. 10.1242/jcs.112045 23720376 PMC3666249

[B30] Kembro J. M. Aon M. A. Winslow R. L. O’Rourke B. Cortassa S. (2013). Integrating mitochondrial energetics, redox and ROS metabolic networks: a two-compartment model. *Biophys. J.* 104 332–343. 10.1016/j.bpj.2012.11.3808 23442855 PMC3552263

[B31] Kim H.-J. Ha S. Lee H. Y. Lee K.-J. (2015). ROSics: chemistry and proteomics of cysteine modifications in redox biology. *Mass Spectrom. Rev.* 34 184–208. 10.1002/mas.21430 24916017 PMC4340047

[B32] Korshunov S. S. Skulachev V. P. Starkov A. A. (1997). High protonic potential actuates a mechanism of production of reactive oxygen species in mitochondria. *FEBS Lett.* 416 15–18. 10.1016/S0014-5793(97)01159-9 9369223

[B33] Kraeutler M. J. Soltis A. R. Saucerman J. J. (2010). Modeling cardiac β-adrenergic signaling with normalized-Hill differential equations: comparison with a biochemical model. *BMC Syst. Biol.* 4:157 10.1186/1752-0509-4-157 PMC2993667 21087478

[B34] Leslie N. R. Bennett D. Lindsay Y. E. Stewart H. Gray A. Downes C. P. (2003). Redox regulation of PI 3-kinase signalling via inactivation of PTEN. *EMBO J.* 22 5501–5510. 10.1093/emboj/cdg513 14532122 PMC213768

[B35] Lim J. B. Huang B. K. Deen W. M. Sikes H. D. (2015). Analysis of the lifetime and spatial localization of hydrogen peroxide generated in the cytosol using a reduced kinetic model. *Free Radic. Biol. Med.* 89 47–53. 10.1016/j.freeradbiomed.2015.07.009 26169725

[B36] Lim J. B. Langford T. F. Huang B. K. Deen W. M. Sikes H. D. (2016). A reaction-diffusion model of cytosolic hydrogen peroxide. *Free Radic. Biol. Med.* 90 85–90. 10.1016/j.freeradbiomed.2015.11.005 26561774

[B37] Linke A. Adams V. Schulze P. C. Erbs S. Gielen S. Fiehn E. (2005). Antioxidative effects of exercise training in patients with chronic heart failure: increase in radical scavenger enzyme activity in skeletal muscle. *Circulation* 111 1763–1770. 10.1161/01.CIR.0000165503.08661.E5 15809365

[B38] Liu S.-S. (2010). Mitochondrial Q cycle-derived superoxide and chemiosmotic bioenergetics. *Ann. N. Y. Acad. Sci.* 1201 84–95. 10.1111/j.1749-6632.2010.05632.x 20649544

[B39] Mahadev K. Zilbering A. Zhu L. Goldstein B. J. (2001). Insulin-stimulated hydrogen peroxide reversibly inhibits protein-tyrosine phosphatase 1b in vivo and enhances the early insulin action cascade. *J. Biol. Chem.* 276 21938–21942. 10.1074/jbc.C100109200 11297536

[B40] Maianski N. A. Geissler J. Srinivasula S. M. Alnemri E. S. Roos D. Kuijpers T. W. (2004). Functional characterization of mitochondria in neutrophils: a role restricted to apoptosis. *Cell Death. Differ.* 11 143–153. 10.1038/sj.cdd.4401320 14576767

[B41] Malinouski M. Zhou Y. Belousov V. V. Hatfield D. L. Gladyshev V. N. (2011). Hydrogen peroxide probes directed to different cellular compartments. *PLoS ONE* 6:e14564 10.1371/journal.pone.0014564 PMC3024970 21283738

[B42] Meier B. Radeke H. H. Selle S. Younes M. Sies H. Resch K. (1989). Human fibroblasts release reactive oxygen species in response to interleukin-1 or tumour necrosis factor-alpha. *Biochem. J.* 263 539–545. 10.1042/bj2630539 2556998 PMC1133461

[B43] Meijles D. N. Fan L. M. Ghazaly M. M. Howlin B. Krönke M. Brooks G. (2016). p22phox C242T single-nucleotide polymorphism inhibits inflammatory oxidative damage to endothelial cells and vessels. *Circulation* 133 2391–2403. 10.1161/CIRCULATIONAHA.116.021993 27162237 PMC6485513

[B44] Meng T.-C. Fukada T. Tonks N. K. (2002). Reversible oxidation and inactivation of protein tyrosine phosphatases in vivo. *Mol. Cell* 9 387–399. 10.1016/S1097-2765(02)00445-8 11864611

[B45] Meyer A. J. Dick T. P. (2010). Fluorescent protein-based redox probes. *Antioxid. Redox Signal.* 13 621–650. 10.1089/ars.2009.2948 20088706

[B46] Moreo A. Ambrosio G. De Chiara B. Pu M. Tran T. Mauri F. (2009). Influence of myocardial fibrosis on left ventricular diastolic function: noninvasive assessment by cardiac magnetic resonance and echo. *Circ. Cardiovasc. Imaging* 2 437–443. 10.1161/CIRCIMAGING.108.838367 19920041 PMC2782553

[B47] Nieborowska-Skorska M. Flis S. Skorski T. (2014). AKT-induced reactive oxygen species generate imatinib-resistant clones emerging from chronic myeloid leukemia progenitor cells. *Leukemia* 28 2416–2418. 10.1038/leu.2014.249 25151958 PMC4262587

[B48] Nieto N. Friedman S. L. Cederbaum A. I. (2002). Stimulation and proliferation of primary rat hepatic stellate cells by cytochrome P450 2E1–derived reactive oxygen species. *Hepatology* 35 62–73. 10.1053/jhep.2002.30362 11786960

[B49] Nishikawa T. Edelstein D. Du X. L. Yamagishi S. Matsumura T. Kaneda Y. (2000). Normalizing mitochondrial superoxide production blocks three pathways of hyperglycaemic damage. *Nature* 404 787–790. 10.1038/35008121 10783895

[B50] Okon I. S. Coughlan K. A. Zhang M. Wang Q. Zou M.-H. (2015). Gefitinib-mediated reactive oxygen specie (ROS) instigates mitochondrial dysfunction and drug resistance in lung cancer cells. *J. Biol. Chem.* 290 9101–9110. 10.1074/jbc.M114.631580 25681445 PMC4423695

[B51] Olsen L. F. Kummer U. Kindzelskii A. L. Petty H. R. (2003). A model of the oscillatory metabolism of activated neutrophils. *Biophys. J.* 84 69–81. 10.1016/S0006-3495(03)74833-4 12524266 PMC1302594

[B52] Padmaraj D. Pande R. Miller J. H. Wosik J. Zagozdzon-Wosik W. (2014). Mitochondrial membrane studies using impedance spectroscopy with parallel pH monitoring. *PLoS ONE* 9:e101793 10.1371/journal.pone.0101793 PMC4091947 25010497

[B53] Park J. Lee J. Choi C. (2011). Mitochondrial network determines intracellular ROS dynamics and sensitivity to oxidative stress through switching inter-mitochondrial messengers. *PLoS ONE* 6:e23211 10.1371/journal.pone.0023211 PMC3150422 21829717

[B54] Parra Cid T. Conejo García J. R. Carballo Alvarez F. de Arriba G. (2003). Antioxidant nutrients protect against cyclosporine A nephrotoxicity. *Toxicology* 189 99–111. 10.1016/S0300-483X(03)00156-2 12821286

[B55] Patetsios P. Song M. Shutze W. P. Pappas C. Rodino W. Ramirez J. A. (2001). Identification of uric acid and xanthine oxidase in atherosclerotic plaque. *Am. J. Cardiol.* 88 A6. 10.1016/s0002-9149(01)01621-6 11448423

[B56] Petty H. R. (2001). Neutrophil oscillations: temporal and spatiotemporal aspects of cell behavior. *Immunol. Res.* 23 85–94. 10.1385/IR:23:1:85 11417862

[B57] Pierce G. B. Parchment R. E. Lewellyn A. L. (1991). Hydrogen peroxide as a mediator of programmed cell death in the blastocyst. *Differentiation* 46 181–186. 10.1111/j.1432-0436.1991.tb00880.x 1655543

[B58] Qu Y. Wang J. Ray P. S. Guo H. Huang J. Shin-Sim M. (2011). Thioredoxin-like 2 regulates human cancer cell growth and metastasis via redox homeostasis and NF-κB signaling. *J. Clin. Invest.* 121 212–225. 10.1172/JCI43144 21123948 PMC3007146

[B59] Recchioni R. Marcheselli F. Moroni F. Gáspár R. Damjanovich S. Pieri C. (1998). Melatonin increases the intensity of respiratory burst and prevents L-selectin shedding in human neutrophils in vitro. *Biochem. Biophys. Res. Commun.* 252 20–24. 10.1006/bbrc.1998.9582 9813139

[B60] Rharass T. Lemcke H. Lantow M. Kuznetsov S. A. Weiss D. G. Panáková D. (2014). Ca2+-mediated mitochondrial reactive oxygen species metabolism augments Wnt/β-catenin pathway activation to facilitate cell differentiation. *J. Biol. Chem.* 289 27937–27951. 10.1074/jbc.M114.573519 25124032 PMC4183826

[B61] Rota C. Fann Y. C. Mason R. P. (1999). Phenoxyl free radical formation during the oxidation of the fluorescent dye 2’,7’-dichlorofluorescein by horseradish peroxidase. Possible consequences for oxidative stress measurements. *J. Biol. Chem.* 274 28161–28168. 10.1074/jbc.274.40.28161 10497168

[B62] Ruiz-Ginés J. A. López-Ongil S. González-Rubio M. González-Santiago L. Rodríguez-Puyol M. Rodríguez-Puyol D. (2000). Reactive oxygen species induce proliferation of bovine aortic endothelial cells. *J. Cardiovasc. Pharmacol.* 35 109–113. 10.1097/00005344-200001000-00014 10630740

[B63] Satapati S. Kucejova B. Duarte J. A. G. Fletcher J. A. Reynolds L. Sunny N. E. (2015). Mitochondrial metabolism mediates oxidative stress and inflammation in fatty liver. *J. Clin. Invest.* 125 4447–4462. 10.1172/JCI82204 26571396 PMC4665800

[B64] Schreck R. Rieber P. Baeuerle P. A. (1991). Reactive oxygen intermediates as apparently widely used messengers in the activation of the NF-kappa B transcription factor and HIV-1. *EMBO J.* 10 2247–2258. 2065663 10.1002/j.1460-2075.1991.tb07761.x PMC452914

[B65] Schwarz K. B. (1996). Oxidative stress during viral infection: a review. *Free Radic. Biol. Med.* 21 641–649. 10.1016/0891-5849(96)00131-1 8891667

[B66] Sedaghat A. R. Sherman A. Quon M. J. (2002). A mathematical model of metabolic insulin signaling pathways. *Am. J. Physiol. Endocrinol. Metab.* 283 E1084–E1101. 10.1152/ajpendo.00571.2001 12376338

[B67] Selivanov V. A. Votyakova T. V. Pivtoraiko V. N. Zeak J. Sukhomlin T. Trucco M. (2011). Reactive oxygen species production by forward and reverse electron fluxes in the mitochondrial respiratory chain. *PLoS Comput. Biol.* 7:e1001115 10.1371/journal.pcbi.1001115 PMC3068929 21483483

[B68] Selivanov V. A. Votyakova T. V. Zeak J. A. Trucco M. Roca J. Cascante M. (2009). Bistability of mitochondrial respiration underlies paradoxical reactive oxygen species generation induced by anoxia. *PLoS Comput. Biol.* 5:e1000619 10.1371/journal.pcbi.1000619 PMC2789320 20041200

[B69] Selivanov V. A. Zeak J. A. Roca J. Cascante M. Trucco M. Votyakova T. V. (2008). The role of external and matrix pH in mitochondrial reactive oxygen species generation. *J. Biol. Chem.* 283 29292–29300. 10.1074/jbc.M801019200 18687689 PMC2570889

[B70] Sharma P. Chakraborty R. Wang L. Min B. Tremblay M. L. Kawahara T. (2008). Redox regulation of interleukin-4 signaling. *Immunity* 29 551–564. 10.1016/j.immuni.2008.07.019 18957266 PMC2631209

[B71] Smith G. R. Shanley D. P. (2013). Computational modelling of the regulation of Insulin signalling by oxidative stress. *BMC Syst. Biol.* 7:41 10.1186/1752-0509-7-41 PMC3668293 23705851

[B72] Smith M. A. Richey Harris P. L. Sayre L. M. Beckman J. S. Perry G. (1997). Widespread peroxynitrite-mediated damage in Alzheimer’s disease. *J. Neurosci. Off. J. Soc. Neurosci.* 17 2653–2657. 10.1523/JNEUROSCI.17-08-02653.1997 PMC6573097 9092586

[B73] Sobotta M. C. Barata A. G. Schmidt U. Mueller S. Millonig G. Dick T. P. (2013). Exposing cells to H_2_O_2_: a quantitative comparison between continuous low-dose and one-time high-dose treatments. *Free Radic. Biol. Med.* 60 325–335. 10.1016/j.freeradbiomed.2013.02.017 23485584

[B74] Swain L. Kesemeyer A. Meyer-Roxlau S. Vettel C. Zieseniss A. Güntsch A. (2016). Redox imaging using cardiac myocyte-specific transgenic biosensor mice. *Circ. Res.* 119 1004–1016. 10.1161/CIRCRESAHA.116.309551 27553648

[B75] Thannickal V. J. Fanburg B. L. (1995). Activation of an H2O2-generating NADH oxidase in human lung fibroblasts by transforming growth factor beta 1. *J. Biol. Chem.* 270 30334–30338. 10.1074/jbc.270.51.30334 8530457

[B76] Valls-Lacalle L. Barba I. Miró-Casas E. Alburquerque-Béjar J. J. Ruiz-Meana M. Fuertes-Agudo M. (2016). Succinate dehydrogenase inhibition with malonate during reperfusion reduces infarct size by preventing mitochondrial permeability transition. *Cardiovasc. Res.* 109 374–384. 10.1093/cvr/cvv279 26705364

[B77] Wang C.-C. Jamal L. Janes K. A. (2012). Normal morphogenesis of epithelial tissues and progression of epithelial tumors. *Wiley Interdiscip. Rev. Syst. Biol. Med.* 4 51–78. 10.1002/wsbm.159 21898857 PMC3242861

[B78] Wang L. Brugge J. S. Janes K. A. (2011). Intersection of FOXO- and RUNX1-mediated gene expression programs in single breast epithelial cells during morphogenesis and tumor progression. *Proc. Natl. Acad. Sci. U.S.A.* 108 E803–E812. 10.1073/pnas.1103423108 21873240 PMC3189061

[B79] Wilmes A. Limonciel A. Aschauer L. Moenks K. Bielow C. Leonard M. O. (2013). Application of integrated transcriptomic, proteomic and metabolomic profiling for the delineation of mechanisms of drug induced cell stress. *J. Proteomics* 79 180–194. 10.1016/j.jprot.2012.11.022 23238060

[B80] Wong G. H. Goeddel D. V. (1988). Induction of manganous superoxide dismutase by tumor necrosis factor: possible protective mechanism. *Science* 242 941–944. 10.1126/science.3263703 3263703

[B81] Wymann M. P. Kernen P. Deranleau D. A. Baggiolini M. (1989). Respiratory burst oscillations in human neutrophils and their correlation with fluctuations in apparent cell shape. *J. Biol. Chem.* 264 15829–15834. 2777766

[B82] Zeigler A. C. Richardson W. J. Holmes J. W. Saucerman J. J. (2016). A computational model of cardiac fibroblast signaling predicts context-dependent drivers of myofibroblast differentiation. *J. Mol. Cell. Cardiol.* 94 72–81. 10.1016/j.yjmcc.2016.03.008 27017945 PMC4861657

[B83] Zhang Q. Pi J. Woods C. G. Andersen M. E. (2009). Phase I to II cross-induction of xenobiotic metabolizing enzymes: a feedforward control mechanism for potential hormetic responses. *Toxicol. Appl. Pharmacol.* 237 345–356. 10.1016/j.taap.2009.04.005 19371757 PMC2696203

